# Becoming nature: effects of embodying a tree in immersive virtual reality on nature relatedness

**DOI:** 10.1038/s41598-022-05184-0

**Published:** 2022-01-25

**Authors:** Pia Spangenberger, Sonja Maria Geiger, Sarah-Christin Freytag

**Affiliations:** 1 Institute of Vocational Education and Work Studies, Technische Universität Berlin, Berlin, Germany; 2 Chair of Consumer Research, Justus Liebig University Gießen, Gießen, Germany; 3 Chair of Human-Machine Systems, Technische Universität Berlin, Berlin, Germany

**Keywords:** Environmental social sciences, Psychology and behaviour, Sustainability

## Abstract

The potential of using immersive virtual reality (iVR) technologies to enhance nature relatedness by embodying non-human beings, such as plants or animals, is only sparsely researched. To contribute to this emerging research field we conducted an experimental study (N = 28) that compared the effects of the viewing condition (iVR or desktop) while embodying a tree on nature relatedness, perspective-taking and, as a control, on perceived immersion. A mixed-method approach employing quantitative and qualitative questions was used. Our results showed that irrespective of condition allocation, the more immersed participants felt in their experience, the greater they reported increased levels of nature relatedness (r = 0.42, *p* < .05). While our quantitative data did yield a difference in immersion levels between the viewing condition (iVR vs. video, t(26) = 2.05, *p* = .05, d = .50) that did not translate into a stronger experimental effect of the iVR condition on nature relatedness (F_Interaction_(1,26) < 1). Regarding perspective taking, no significant differences between both groups emerged in the number of users who self-reported having fully taken on the perspective of the tree, (χ^2^(1) = 2.33, *p* = .127). However, only participants from the iVR group described their experience from a first-person perspective, suggesting a higher level of identification with the tree. This matches the observation that only those participants also reported self-reflective processes of their own role as a human being towards nature. Our results support previous research suggesting that experiencing nature via immersive VR in itself does not seem to suffice for creating an effect on nature relatedness. However, we observed that a higher perceived level of immersion for participants experiencing the embodiment of a tree in the iVR condition provoked reflective processes on one’s own role towards nature more strongly. We discuss the role of immersion and further factors to explain these differences and suggest steps for future research settings to help understand the beneficial potential of using immersive VR for nature relatedness.

## Introduction

One of the most pressing issues of the twenty-first century is to find and implement countermeasures to the ongoing climate crisis and destruction of nature. While technical solutions are being developed at a fast pace, successful implementation depends not only on the availability of technology but on creating an awareness about mechanisms of sustainable development and on transferring this awareness into actual actions on the individual level. Besides efficiency and consistency as promising technology-based strategies, a person’s relationship towards nature has been argued to be a predicting factor for sustainability-oriented behaviour within a sufficiency strategy^1^. Thus, it is vital for education for sustainable development that this relationship is nurtured and supported^1,2^. While a cognitive understanding of the impact of one’s daily actions on nature is the intellectual base for finding new solutions^2^, they will only be implemented if a corresponding motivation is present. The role of other species and natural systems and their importance for the ecosystem have to be understood in order for people to be able to reflect on their relatedness towards nature^1,3^. Thus, it requires methods that address the affective dimension, break through previous habits, and train the ability to act^4,5^. In the concept of Education for Sustainable Development perspective taking is argued as an approach to foster understanding and reflecting one's relationship towards others (cognitive dimension), and by that, relating to it (affective dimension)^6^. Based on the idea of “perspective transformation” by Mezirow^7^, the experience of taking on someone else’s role, by methods as such as role-play, is discussed as a promising method to reflect on one's own role in climate change, understand one's personal impact on climate change and promote the ability to relate to others^8^. Mayer and Frantz^1^ argue that a feeling of connectedness to nature leads to a stronger concern for nature and can invoke action such as pro-environmental behaviour. Previous studies have shown that direct exposure to nature can strengthen a feeling of nature connectedness or nature relatedness^1,9–11^. It can also influence environmental knowledge, attitudes, or behaviour^12–15^. In line with environmental psychology^16,17^, we define nature as ‘…a broad category of natural environments and features of those environments, such as single trees or plants’ (van den Berg et al.^17^, p. 57). This understanding includes images of nature in the form of videos, films, or other imagery.

However, due to urbanization, it has been observed that more people live removed from nature, e.g., natural environments such as forests, and those have a low concern for nature^15^. Today, 82 per cent of the North American population, 81 per cent of the Latin American and the Caribbean population, and 74 per cent of the European population live in urbanized areas. This urbanization trend is predicted to grow from 55 per cent to 68 per cent of the worldwide population by 2050^18^, with the result that the accessibility of nature, and thus the ability to experience it in person, is reduced. It then becomes harder to see oneself as part of a natural ecosystem, which in turn may lead to less concern for nature and thus less pro-environmental behaviour. At the same time, traveling to especially highly valuable ecosystems in terms of biodiversity and greenhouse gas capture as e.g., the Amazon, is neither feasible to provide a large number of humans with this kind of experience nor without risk for the local ecosystem. This raises a vital question: How can the processes of reflecting on oneself as part of the natural ecosystem be supported for those without access to natural environments, be it due to urbanization or other restrictions? The positive effects of being exposed to nature are not limited to experiences in real-world nature settings, as Meidenbauer et al.^19^ demonstrated. According to their study, even the act of simply looking at an image of nature or virtual environments depicting nature could achieve similar results. However, Zelenski et al.^20^ found that exposure to nature via video-watching can promote ‘…greater willingness to engage in environmentally sustainable behavior’ (Ref.^20^, p.24). These effects become more pronounced the more users perceive the experience as a real, personal one.

New solutions to provide such real, personal experiences of distant biospheres might be offered by modern immersive virtual reality (VR) technology. Thanks to highly developed technology, virtual sights of nature can be experienced with a 360°-degree angle (e.g., Nature TreksVR). Putting on a Head-Mounted-Display (HMD), users can go beyond simply looking at a landscape in front of them. Instead, users can be completely surrounded by it. Hu-Au and Lee^21^ argue that immersive VR technologies offer increasing engagement, provide interactive, action-oriented, affective, and empathetic experiences, and can serve as an ‘arena for visualising’ (Ref.^21^, p.216). Individuals can take on someone else’s perspective, close the time gap between action and consequences, get interactively involved, receive direct feedback on decisions and behaviour, see consequences, foresee future climate change scenarios, and experience sensory stimulations that can have a strong impact on affections^22–26^. The assisting role and the success of technological components in creating a convincing and captivating experience can be subsumed under the term “immersion”^27^, while the engagement of multiple sensory channels has been coined “sensory immersion”^28^. Both definitions suggest that the perceived presence is influenced by the level of immersion provided by a virtual application, and the technological components used to experience the content. This in turn influences the motivation to transfer what was learned into actions. Immersive VR technologies are defined as technologies that immerse the user as much as possible in the virtual experience, especially via the use of HMDs^29^, which allow the users to translate their natural head movements into camera movements within the virtual environment, providing a higher level of immersion compared to watching videos or pictures via a desktop screen^30^. Immersive VR shows promising potential to reduce the gap between virtual representations and real-life experiences, which are vital to fostering behavioural change. Positive effects of VR applications on motivation, knowledge, engagement, task performance, and long-term retention have already been observed in the context of learning^31–35^. Today, immersive VR technology has evolved to a point where users can enter immersive artificial environments via HMD comfortably from their own living rooms^30^. Worldwide, the demand for VR headsets is forecasted to reach USD 62.1 billion by 2027^36^. Side effects such as motion sickness have become more preventable via the appropriate design of the virtual environments or by accustomization^37^. Devices have become affordable, do not necessarily require access to high-end PCs, and can be used with a smartphone (e.g., Google Cardboard, the Oculus Quest, Valve VR, or the HP Reverb G2). This development is a prerequisite for bringing the proverbial mountain to the prophet: It allows researchers to provide the most immersive portal to nature experiences to people who are unable to have these encounters in-person.

However, there are still only limited numbers of virtual nature applications on HMD available, and valid research results for the use of these applications in the various fields of the Sustainable Development Goals (SDGs) are still in the nascent stage^26^. So far, there are only few data on the cognitive, socio-emotional, or behavioural effects of immersive VR technology applied to environmental awareness. Studies on immersive nature experiences in VR have investigated effects on mood^23,38^, physical engagement^39^, green product consumption^40^, interest^26^, pro-environmental behaviour^22,25,41^, and nature relatedness^41^. In the majority of the studies, participants have been exposed to 360° videos of nature via desktop or HMD. Ahn et al.^22^ showed that climate-change-related applications experienced via a HMD ‘…can be strong enough to transfer into the physical world to modify behaviour’ (Ref.^22^, p. 85). Filter et al.^26^ let students experience 360° videos about the life of wolves via HMD, showing that immersive technology can foster interest in nature experiences. Klein and Hilbig^25^ exposed participants to nature videos of trees or birds and compared it to conditions of watching videos about social interactions or urban environments. The authors observed that watching videos of nature destruction can have a stronger impact on pro-environmental behaviour compared to experiencing a video about actual intact nature. Soliman et al.^41^ investigated effects of artificial nature videos vs. real nature videos on nature relatedness and pro-environmental behaviour. As one of the results, the authors observed that watching videos of nature can foster nature connectedness irrespective of the technology used (immersive VR vs. desktop screen). Mostarejan et al.^38^ showed that watching 360° videos of a forest via HMD has a stronger effect on mood compared to looking at pictures of a forest using HMD.

The beneficial effects of immersive VR on inter-human relationships and cognition seem to be reproducible for the interaction with the impersonal ‘other’, such as nature. Immersive VR, with its typical display mode of exploring experiences from a first-person perspective, facilitates taking on the perspective from which the experience was filmed or created. This allows the experience of embodying the portrayed agent. This is a prerequisite for the learning transfer of applications set in the context of sustainable development. Seeing the potential of what effect the mere visual experience of virtual representations of nature can have on nature relatedness, a more immersive experience such as embodiment of nature could increase the impact even further. Available VR applications can include experiences that play with what it means to see the world from another’s eyes. Experiences such as ‘The Machine To be Another’ from AnotherLab have provided insights into how swapping perspectives and embodying another person can be used to train empathy^42^. The beneficial effects can far surpass momentary affections: By swapping perspectives with that of female victims of domestic abuse, male offenders have not only reported increased levels of empathy towards victims but experienced the long-lasting effect of being able to better judge the emotions of others^43^. While the potential of embodiment, body-swapping, or body-ownership of a human in VR has been examined in various research contexts, research on non-human embodiment such as embodiment of an animal, a robot, or a plant is still in its early stages^44–47^. Ventre-Dominey and colleagues^46^ have examined the effects of embodying a robot on its acceptability. The authors observed that taking on the perspective of a robot can increase its likability as long as one´s own body movements match the movements of the robot in VR. Oyanagi and Ohmura^47^ focused on the effect of embodying a bird on anxiety about heights. The authors could report a decrease in self-reported fear of heights. As of now, to our knowledge, there are only two studies that focus on embodiment and its effects in the context of nature relatedness^44,45^. Markowitz et al.^45^ conducted a study comparing non-human embodiment (a coral) and human embodiment (a scuba diver) stating that ‘…the more that people reported being attuned to the virtual environment in the post-test, the more they learned in immersive VR, felt connected to nature, and reported environmental concern’ (Ref.^45^, p. 10). In several experiments by Ahn and colleagues^44^, effects on nature connectedness by being a coral and a cow were measured by comparing the VR experience via HMD to watching a desktop video. The authors observed that embodiment of a virtual other, was crucial for a high degree of connectivity to nature. They argue that embodiment in VR can foster especially the potential of perspective taking.

In our study, we transfer and expand the ongoing embodiment research to non-human and non-animal agents, and systematically compare the effects of embodying a tree between a standard viewing condition and an immersive VR (iVR) condition that displays the experience with a HMD and features the option to make small branch movements via controllers on nature relatedness. While perspective taking in itself is associated with favourable attitudes, the immersion via HMD in a virtual environment has been shown to be the determining factor of the occurrence of attitude change^48^. Therefore, a combination of perspective taking supported by embodiment through iVR is a promising approach to explore the transfer to a non-human, non-sentient entity in the context of climate change and nature relatedness. Thus, the aim of our study was to investigate whether experiencing the embodiment of a tree via iVR fosters a) a feeling of immersion, b) relatedness with nature, c) perspective-taking, and d) reflection on the relationship between humankind and nature. Taken this under consideration, we understand our study as continuing the conversation on how embodiment in VR technology can foster nature relatedness.

We carried out an experimental study with 28 participants in a 2 × 2 × 2 between-subjects design with *condition* (iVR vs. video watching) and *ending* (negative vs. positive) as between-subject factors and *time* (pre-post measurements) as a within-subject factor. Both experiencing conditions differed as follows: the iVR experience allowed for free head movement, creating the ability to look around freely. Additionally, hand-held controllers translated the users’ arm movements into a slight movement of the tree’s branches. The video desktop screen condition displayed a fixed orientation of the view and did not include the interactive element of branch movement. This decision was made based on the technical limitations of making the experience which has been developed for iVR accessible via desktop screen.

Three dependent variables (perceived immersion, nature relatedness, perspective-taking) were measured with a questionnaire that asked additional open questions to tackle further reflections on the experience of embodiment. Participants were randomly assigned to one of four conditions (iVR vs. video watching; positive vs. negative ending). As positive vs. negative endings were not of focal interest and did not yield different results, we report hypotheses and results for the factor experience condition only. Thus, the following hypotheses were tested:

- H1: Experiencing the embodiment of a tree via iVR is perceived as more immersive than watching the experience as a video on a desktop screen.
- H2: Experiencing the embodiment of a tree via iVR leads to higher levels of nature relatedness than watching the experience as a video on a desktop screen.
- H2b: Perceived immersion levels are associated with an increase in nature relatedness.
- H3: Experiencing the embodiment of a tree via iVR facilitates more perspective-taking of the tree compared to watching the experience as a video on a desktop screen.

We were also interested in exploring the subjective experience of participants concerning the reflection of their own relationship with the tree, assuming that the experience of embodiment of the tree in iVR initiates a stronger process of self-reflection than when watching a video of the same experience as a video on a desktop screen (H4). We therefore added open questions, described in measures. By the explorative combination of qualitative and quantitative data, we hope to enrich the discussion about the effects on embodiment in iVR as a tool to foster perspective-taking as one relevant goal of education for sustainable development by means of reflecting on the role of other living beings on this planet^2,6–8^.

## Materials

**Figure 1:**
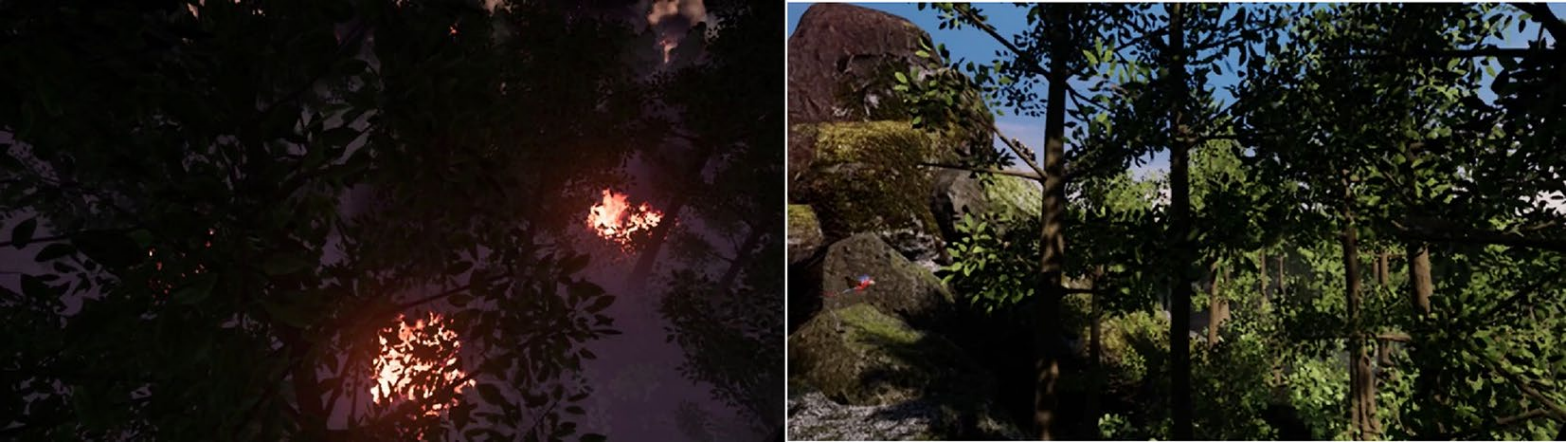
View from the perspective of the tree during the forest fire (to the left) and during daylight (to the right).

The application ‘Tree’, available for HTV Vive, was used as stimulus material. ‘Tree’ has been conceptualized and realized by a group of designers and researchers at MIT in collaboration with film directors^49^. The application used has been screened as a film at over more than 60 festivals worldwide and won multiple awards (https://www.treeofficial.com/). Using a head-mounted-display, the user takes on the view of a tree in the Amazon rainforest. The user experiences the birth of a tree and its life in a fixed chronological order. The growth is portrayed, first underneath the earth at the beginning, then up until the final height of the tree. This experience is displayed from a first-person point of view, giving the impression of being the tree. Upon reaching the full height, night falls. A fire starts in the far distance, accompanied by drum sounds. The fire approaches and neighbouring trees catch on fire. Right before the portrayed tree catches on fire, too, the perspective shifts to a third-person view of the tree and the experience ends. Two adaptations were made to the material: First, a second version with a positive ending was created by having the original experience which lasts for 6:48 min, stop at the 4:40-min mark upon nightfall, omitting the forest fire. Second, to make the experience available as a video watching condition, a screen recording of the iVR experience was created for both the positive and the negative ending (see Fig. 1).

To develop the video version of the tree’s experience, we recorded a game play of a volunteer in iVR via HMD prior our experiment including turning the head movements for close-up views of targeted objects (e.g., birds, monkeys). The course and content of the experience was identical in both experiencing conditions, and participants experienced the application either in 4:40 min (not including forest fire) or in 6:48 min (including forest fire) in one of the viewing conditions (iVR or video watching). Also, in both conditions, a background sound of animals in the Amazon was audible as part of the soundscape of the application using the same sort of headphones. The difference between conditions in our experiment was that in iVR, participants were able to slightly move the tree’s branches via controllers. We understand this movement as extending part of the embodiment experience in iVR via HMD. Thus, the conditions were completely comparable with exception of the ability, when used, to move the branches and to freely look around in 360 degrees during the fixed course of events (birth, life, and end).

## Methods

### Participants

28 students participated in the study (14 = male; 14 = female). Participants were between 22 and 35 years old (M = 26.32; SD = 3.1). The participants volunteered to be part of the research project and were not compensated for their participation. The study was carried out in accordance with the guidelines of the German Psychological Society provided as self-assessment-tool by the Ethics Committee of the Department of Psychology and Ergonomics of Technical University of Berlin in accordance with the Declaration of Helsinki (2013) with written informed consent from all subjects. Participants of the study were informed about the content of the experiment, possible adverse effects of exposure to the virtual reality application, the anonymization procedure, and data usage. Participants were informed about the voluntary nature of the study and were told that they were able to withdraw from the experiment at any point without need to state any reason and without any consequences. Furthermore, before the study, participants were asked about prior experiences with motion sickness, and if they had experienced motion sickness, explicitly asked to verify their participation. They could request the deletion of their data within 7 days of their participation.

### Procedure

**Figure 2:**
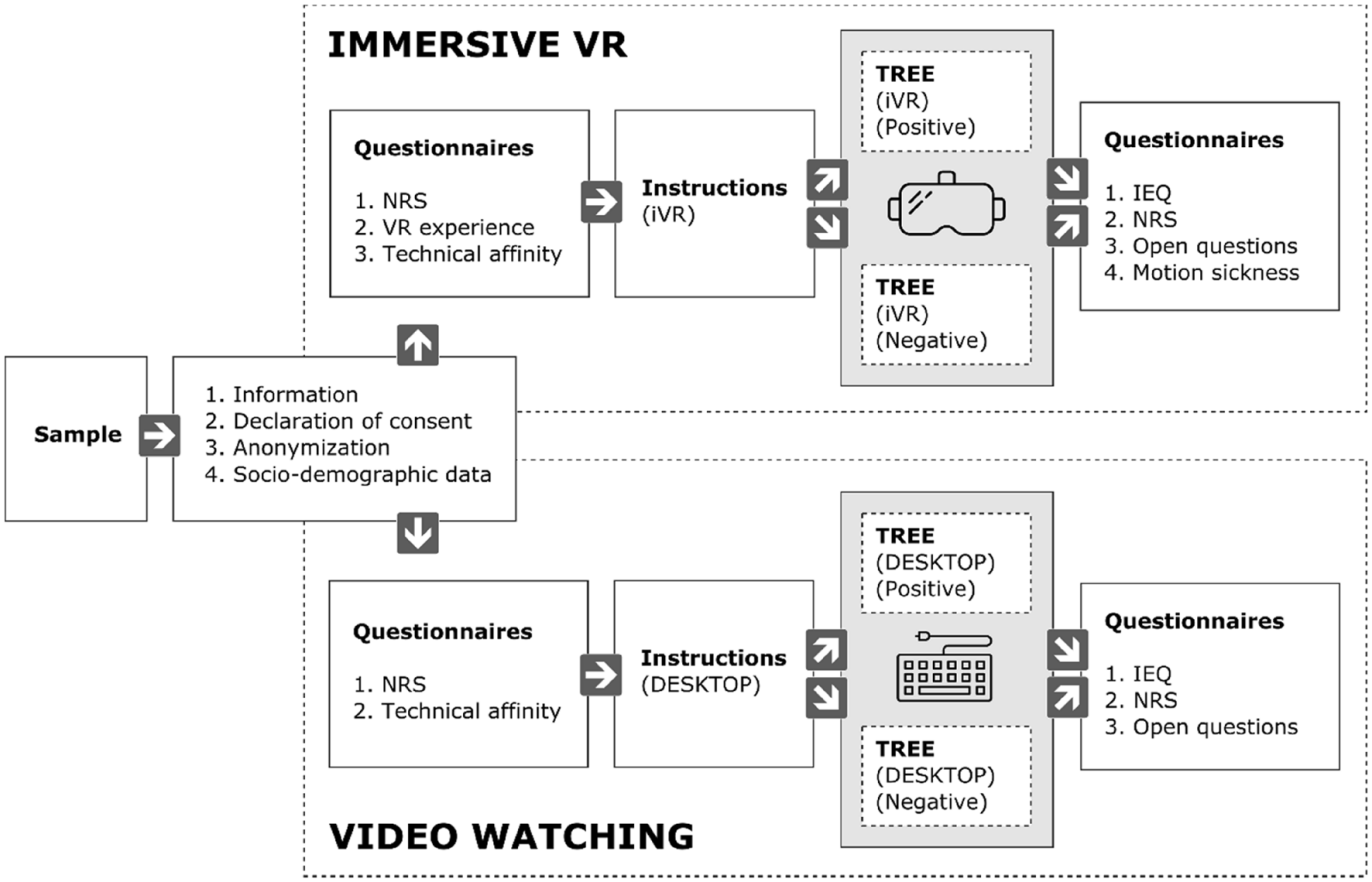
Experimental procedure for the conditions. Own illustration, adapted from final report of students^50^. IEQ = Immersion Experience Questionnaire; NRS = Nature Relatedness Scale.

Participants were randomly assigned to one of the conditions (iVR or video watching on a desktop screen and positive or negative ending). Each participant was greeted by the assigned experiment conductor. Participants were then asked to read a short description about the experiment and signed the consent form. Afterwards, they were asked to fill out our pre-test questionnaire. If selected for the video watching condition, participants started to watch the experience via video on a desktop screen. If selected for the iVR condition, participants were guided to the middle of the VR tracking area and received controllers and headphones (see Fig. 2). In the iVR condition, participants started in the so-called ‘credits room’ of the Tree application to get used to the visuals and the VR environment. After that, the actual Tree experience was started. After the experiment, in both experimental conditions, participants completed the post-test questionnaires containing quantitative and qualitative questions to describe their experience during the experiment in detail. Comments or difficulties during the experiment were documented by the experimenters.

### Technical equipment

For the immersive VR condition, an HTC-Vive (MV HTC LHR-7433AF4C) including HTC-Vive controllers (MV HTC LHR-FFA61F42 & MV HTC LHR-FF663B42) was used as a means of display and interaction, respectively. The sound output was transferred via on-ear headphones in both conditions. A desktop PC with Windows 10 and a 24-inch monitor (NVIDA GeForce GTX 1060) with a resolution of 1920 × 1080 pixels was used for the video watching condition.

### Measures

The following scales and questions were used within the study:

#### Socio-demographic data

The questionnaire contained items on socio-demographic data such as age, gender, prior experience with VR applications (one item, ‘Do you have any previous experience with VR?’), and technical affinity (one item, ‘How interested are you in using electronic devices such as computers, tablets?’).

#### Perceived immersion

The Immersive Experience Questionnaire (IEQ) has been classified as a valid instrument to measure immersion in digital games^51–56^. The IEQ aims to assess the notion of immersion and defines immersion as a concept that contains five factors (cognitive involvement, emotional involvement, real world dissociation, challenge, and control). This classification is in line with the definition of immersion and immersive VR by Slater^30^ and Kim and Biocca^28^ which we employ. The short scale contains 18 items (5-point Likert scale from 1 = I strongly disagree to 5 = I strongly agree, e.g., ‘I had the feeling of being separated from the real world’; Bentler’s Omega Ω = 0.80; 3 items reverse-scored). To our knowledge, there is no validated German version, thus, the questionnaire was translated into German.

#### Nature relatedness

The extent to which people feel related to nature was measured with the Nature Relatedness Scale (NRS). The NRS contains affective, cognitive, and experiential items asking about one’s relation to nature^10^. The German version^57^ of the Nature Relatedness Scale- (NR-6) by Nisbet and Zelenski^58^ contains 6 items (5-point Likert scale from 1 = I strongly disagree to 5 = I strongly agree, Bentler’s Omega Ω = 0.78_pre_/0.85_post_). The NR-6 is based on a longer version of the NRS with 21 items^9^, and the NR-6 (hereafter referred to as the NRS) correlates strongly with the longer version^58^ (Bentler’s Omega Ω = 0.78).

#### Perspective-taking

To explore a deeper understanding of the embodiment experience of the tree, participants were presented four open questions, a) how did you like the application Tree? B) could you take on the perspective of the tree, and if not, please specify your experience, c) did you notice the presence of any other animals in the application, d) did the experience evoke any emotions in you? If yes, please describe them in your own words.

#### Motion sickness

Participants assigned to the iVR group were controlled for motion sickness using the Virtual Reality Sickness Questionnaire (VRSQ) by Kim et al.^59^ containing 9 items. The items describe different symptoms (e.g., headache, tiredness) to be rated on the strength of their occurrence during the experience (1 = not at all to 4 = strong).

## Results

### Effects on perceived immersion (Hypothesis 1)

A t-test for independent samples (t(26) = 2.05, *p* = 0.05, d = 0.50) revealed that participants who experienced the embodiment of a tree in iVR perceived the experience as more immersive (M = 3.35, SD = 0.53) than those who were watching a video on a desktop screen (M = 2.97, SD = 0.62).

### Effects on nature relatedness (Hypothesis 2)

A two-factorial ANOVA for nature relatedness revealed neither a main effect of time (F(1, 26) = 1.95, *p* = 0.18) nor of condition (F(1,26) < 1). Presented in Table 1, the descriptive statistics reveal that neither group increased their nature relatedness substantially, and the expected stronger effect for the iVR group was also not observed, as indicated by the lack of interaction between time and condition (F(1,26) < 1).

**Table 1:** Descriptive statistics for immersive experience and nature relatedness, pre- and post-experiment.

	Condition	Pre		Post		n
	iVR versus video watching	M	SD	M	SD	
Immersive experience (IEQ)	iVR			3.35	0.53	14
	Video watching			2.97	0.45	14
Nature relatedness (NRS)	iVR	3.12	0.64	3.17	0.82	14
	Video watching	3.30	0.83	3.45	0.85	14

1 = 5-point Likert scale (1 = strongly disagree to 5 = I strongly agree).

### Relation of perceived immersion and nature relatedness increase (Hypothesis 2b)

**Figure 3:**
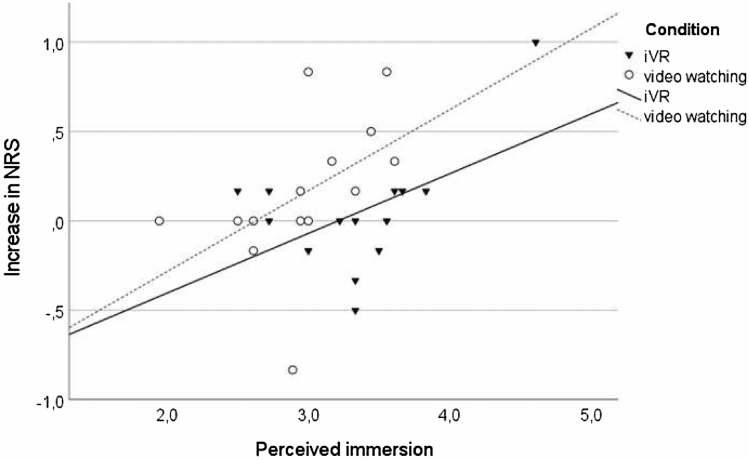
Correlation of perceived immersion and increase in nature relatedness. *Note*: Data points correspond to scale means (perceived immersion) and post–pre delta values of scale means (increase in nature relatedness) for each participant.

To further explore the role of immersion in strengthening nature relatedness, we ran a correlation analysis of the scale means of reported immersion and the delta values of post–pre measures for the nature relatedness of each participant. A significant association (r = 0.42, *p* = 0.034) was found as presented in Fig. 3. This means that irrespective of their condition allocation, the more immersed people felt in their embodiment experience, the greater was their increase in nature relatedness.

### Effects on perspective-taking (Hypothesis 3)

10 out of 14 of participants (71%) in the iVR condition felt they were taking on the tree’s perspective, compared to 6 out of 14 participants (42%) in the video watching condition. This distribution did not differ significantly from an equal distribution (χ2(1) = 2.33, *p* = 0.127).

Taken together, our quantitative results show that a) while on average the iVR group felt more immersed (see H1), b) the video watching group showed a stronger nature relatedness increase per immersion unit (higher slope) and c) across all participants, there was a medium-sized correlation between perceived immersion and increase in nature relatedness (see H2b).

### Qualitative results

#### Exploration on reflective processes

Participants who could not take on the tree’s perspective mostly mentioned reasons such as the ‘*movements of the tree didn’t feel natural or real*’ (4x), followed by individual reasons such as ‘*the tree itself did not make any sound*’ (1x), ‘*animation was not real enough*’ (1x), ‘*I could not see the tree’s trunk*’ (1x), ‘*a tree perspective was too abstract to me*’ (1x), ‘*the zoom-in function was too confusing’* (1x), and, ‘*the desktop screen was too small*’ (1x). The response to the open question of how the Tree application was perceived showed that 24 out of 26 participants reported a very a positive impression of the application, described in terms such as ‘*fascinating*’ (2x), ‘*exciting*’ (3x), ‘*powerful*’ (1x), ‘*beautiful*’ (3x), ‘*very interesting*’ (7x), and ‘*great*’ (3x). At the same time, participants described in detail how their impressions were perceived. For instance, one participant in the iVR condition perceived the application as fascinating because he/she ‘*felt affected by the fate of the tree*’. Other participants (desktop condition) described the experience as beautiful because they felt a sense of ‘*cosiness*’ and ‘*security*’ as well as ‘*a feeling of wanting to protect nature*’. Two participants reported no feelings at all besides being amused and astonished that ‘*The tree grew damn fast within one day*’ (desktop condition). Another participant also referred to the fast growth of the tree as they reported that they ‘*felt nothing but curiosity*’ (iVR condition). Answering the question how the experience was generally perceived, especially participants of the iVR condition also indicated experiencing an elevated sense of presence while in VR. For instance, participants reported ‘*Great. The growth of the tree and the exploration possibilities are impressive, and you have the feeling of being there completely’*, and ‘*I thought it was great, it’s crazy how close to reality it is. You really have a little bit of the feeling of really being there*’, as well as ‘*I felt like I was in the rainforest*’. Two participants indicated a perspective change by reporting their experience out of the tree’s perspective, using first person pronouns: ‘*My body became taller and taller*’, and ‘*I am part of the forest and also have a great role in nature as a tree.*’

Answering the qualitative question about which emotions, if any, were involved (n = 27), eight participants described feelings of relaxation (5 × video, 3 × iVR), four participants reported feelings of concern or stress (2 × video, 2 × iVR), and five participants reported sadness (2 × video, 3 × iVR) which was equally distributed between the positive and negative endings. While five participants of the iVR condition (4 × negative ending, 1 × positive ending) explicitly reported a self-reflection of their own role as a human being towards nature such as ‘*You immediately dealt with it in your head and recognized the beauty of nature. I feel a little bad now that we humans are destroying nature like this*’, and ‘*At first, I felt excitement and curiosity. Then wanderlust at the starry sky, followed by consternation at the start of the fire, and then, the feeling that we as a society are to blame for it*’. Another participant said he/she felt ‘*Sadness about what fires, partly caused by humans, cause, and especially with regard to the current situation in the Amazon, shocked*’. The answers to the open question ‘Which animals were noticed during the experience’ varied in content, but participants over all conditions reported that they had perceived a variety of animals, such as birds, a tiger, a monkey, insects, or butterflies.

### Further results

Overall, participants of the sample were very interested in technical devices (M = 4.61, SD = 0.57), and 22 participants of the sample (78.57%, N = 28) already had experience in immersive VR using HMD applications. On average, participants would recommend the application to others (M = 6.5, SD = 2.68). Participants reported almost no signs of motion sickness (M = 1.37, SD = 0.23).

## Discussion

Our study provided results that are partly in line with previous studies regarding embodiment and nature relatedness in immersive VR. Firstly, the embodiment experience of a tree via iVR is perceived as more immersive compared to watching it as a video on a desktop screen (H1). Against our presumptions, the embodiment experience fosters nature relatedness neither generally nor respective of condition allocation (H2). Crucial for a rise in nature relatedness seemed to be the perceived feeling of immersion (H2b) and not the viewing condition (iVR vs. video) per se. These results are in line with Soliman et al.^41^. Although Soliman et al. compared watching videos via HMD and desktop screen, the authors also observed that immersive VR technology itself does not automatically lead to higher nature relatedness. It can be assumed that immersive VR technologies are not yet at the ultimate level of what is technically possible, and some technical issues come with their use that some features (e.g., bucking, lighting conditions, individual expectations) might counteract immersion. Ruling out technological aspects, individual differences in expectations towards the iVR technology as well as the individual readiness to immerse are likely to affect the outcome and, while not accounted for in this study, should be further investigated in future experiments. As Ahn et al.^22,44^ and Ventre-Dominey et al.^46^ point out, interactivity and movements of the person’s own body resembling the virtual body play a significant role in fostering perceived embodiment, and in the case of nature connectedness or nature relatedness, might be more relevant than perceived presence^44^. Furthermore, our quantitative data did not support the hypothesis that embodiment of the tree via iVR facilitated perspective-taking (H3). As no differences between conditions emerged, technological immersion factors can be regarded as playing a secondary role in explaining the readiness to take on the embodied perspective, suggesting that an investigation of personality traits and individual factors might be worthwhile.

Contrary to the quantitative results, the qualitative data suggest that embodiment of nature can be a beneficial approach to initiate a reflective process on the role of other species as part of our eco-system. Participants reported a high amount of perceived realism, of presence, and felt an impact on their emotional state, such as subjective feelings of connectedness with nature, in line with previous research^22–25^. Only those participants in the iVR condition self-reported reflecting on the relationship between humankind and nature, as indicated by statements referring to their impact as a human on the tree’s fate or on themselves as being the tree. The reported subjective data shows how the experience of being one single tree can transcend the individual level and evoke reflective processes on a more universal level, relating to the distant biosphere of the Amazon rainforest and nature as a whole. The selection of the distant and unfamiliar setting of the biosphere in the Amazon was intended to reflect the unfamiliarity of a future target group for such interventions with the ecological environment they are supposed to experience. We assumed that most, if not all, of our participants have not experienced such a setting in person, allowing us to exclude a familiarity bias as much as possible. However, we did not address the familiarity with the environment separately, which is something to be added in future studies.

In our study, there were some limiting factors to taking on the perspective of another species. 29% of participants in the iVR condition and 58% in the video watching condition reported not having taken on the perspective of the tree (a total of 43%). The question of why perspective-taking did not happen in these cases remains open. Technical conditions in both experimental groups might have contributed to the limited perspective-taking. Qualitative answers clearly showed that features of the display method were decisive in whether or not a person made the leap into the tree’s perspective (e.g., ‘*the screen is too small*’). Between conditions, however, not only did the display technology differ, but also the extent to which participants were able to explore the virtual environment. The resemblance to the own body was provided by giving the participants in the iVR condition the opportunity to slightly move the virtual branches with the handheld controllers, creating an analogy to their real arms. This amount of movement was small and seemed to not have sufficed to impact the amount of perceived immersion between both groups in a significant way. However, the qualitative data suggests a higher rate of perspective-taking in the iVR group, indicated by statements such as ‘*My body became taller and taller*’, to which the higher number of immersive features might have contributed. Users typically watch videos without further means of interaction, and without an option to choose different orientations of views. Users with access to an HMD do experience a higher level of possible interactivity. In regard to the question of how we can transport the experience of nature to those who do not have access, any findings that show a difference between those conditions would illuminate whether ‘traditional’ experiences via video are enough to provoke reflective processes, or whether additional levels of immersion are needed and additional costs are justified. Adding more components that address more senses such as touch or smell could create an even more immersive experience that might be able to increase a full-sense immersion via immersive VR technology. This is especially vital in regard to the implementation and widespread distribution of such experiences – what equipment needs to be provided? In future studies, it would be of interest to enhance the levels of possible interaction and sensory channels, and systematically vary them in order to isolate the role of interactivity and also the role of the direct translation of one’s own body movements to the virtual self.

Furthermore, taking on the tree’s perspective in immersive VR might have depended on the individual’s tendency for immersion^60^. Another factor might be the discrepancy between the person’s own body and the body of the embodied agent, as Ahn et al.^44^ pointed out. In their experiment, participants inherited the virtual body of a cow to foster interconnectedness with nature. The authors state that ‘…it is important that the individual feels that he or she actually owns the body of the animal.’ (p.411). The fact that users had to embody a tree, might have been difficult because a tree’s body differs from the human body. Against this background it might have been challenging to create a feeling of body ownership regarding the tree. A further factor on influencing perspective-taking can be individual’s prior VR experience. 78% of participants had already used an iVR HMD prior to our experiment. Thus, the acquired results reflect the experiences of the content itself, rather than the experience of the new technology. For instance, the expectations for graphic illustrations and visualizations may have been higher compared to users with less experience in the use of technical devices and VR specifically, including statements such as ‘animation was not real enough’ or ‘my body movements did not match the movements of the tree’. In the context of perspective-taking, it would also be of interest to compare different agents that participants have to embody. For instance, in a previous study, Markowitz et al.^45^ compared being a coral with being a scuba diver in immersive VR regarding its effects on nature connectedness, but found no significant differences. To our knowledge, this is the only study of this kind comparing two different embodiment conditions. Following this, in future experiments, additional points of view concerning the tree’s fate could be considered to examine its effects on nature relatedness, such as a bystander’s perspective, a third person’s view, or another person’s view, e.g., a fire fighter. The questions of which entity should be embodied and how much interaction the embodiment experience should provide could be explored in future experiments.

Despite the short amount of time spent engaging with the virtual experience, participants of both conditions described a wide range of experiences, with some participants expressing fascination or deep concern for nature. However, the quantitative results reflect this limited exposure: the expected overall effect sizes were small. Thus, to gain a deeper understanding of the individual experiences, we reported qualitative data which yielded further insights. We did not account for the subjective readiness to immerse into the experience in this study, which should be addressed in further studies. Due to the low number of participants (n = 28), the experiment should also be replicated again with a larger samples size to have enough power to detect differences in nature relatedness and perspective-taking. An investigation of the optimal duration of embodiment applications would also be helpful to maximize the impact while keeping the time investment for each potential user at a minimum.

Regarding our measurements, future studies should include different questionnaires to examine the relationship towards nature, because quantitative and qualitative answers were inconclusive with the tools we used. Furthermore, the ad hoc translation of the IEQ scale into German has not been validated by a back translation into the original English. This might have reduced the validity of items and should be addressed in a further study. Additionally assessing physiological data in relation to the experience would provide valuable insights into the users’ experience at each point during exposure to the application, further pinpointing scenes of interest and disruption. Indicators of stress levels, such as body temperature, heartbeat, or cortisol measures before and after the application, and gaze data over the whole duration could further support the understanding of the impact of experiencing the embodiment of a representative of a non-human species. Furthermore, we focused on assessing users’ rating on short term effects by taking measurements right before and after the intervention. Further research projects should focus on long-term changes in concern for the environment as well as on behavioural indicators of these effects resulting in action. It also has been argued for a stronger focus on belief systems instead of emotions when investigating the relationship towards nature^45^. Recording potential changes in behaviour over a longer period after the experience had ended was not within the scope of the experiment. However, Rieckmann^6^ and Qablan^8^ argue, reflective processes can promote later action-oriented behaviour towards climate protection. Such processes were reported by participants of the iVR condition within qualitative data, but not within the video watching group, suggesting a higher potential for embodiment via immersive VR. Finally, in our study, embodiment was understood as a given feature set by the immersive VR technology via HMD. In future studies, it might be of interest to measure self-perception of the embodiment experience using a reliable scale. An appropriate choice would be a scale developed by Slater et al.^61^ to measure embodiment of another human. This scale has already been adapted to the case of virtual animal embodiment by Ahn et al.^44^ and could be adapted for applying it to embodiment of a tree.

In sum, our results contribute to the discussion of how embodiment in immersive VR can be extended to other species, even those perceived as non-sentient. We hope to provide further ideas for examining the role of perspective-taking in fostering nature relatedness with the help of immersive VR. Based on our findings, immersive VR and videos alike can provide personal experiences of nature from a first-person perspective for humans who lack easy access to such settings in the real world. Embodiment has been shown to be a promising tool of taking on someone else’s perspective. Our results suggest that the majority of users are able to take on a perspective of a tree, an organism not usually considered from a first-person point of view. However, we could show that not all users took on the perspective easily, and, in line with Ahn et al.^44^, immersive VR technology in itself might not be enough to promote nature relatedness without taking individual factors into account.

In conclusion, we state that HMDs play a central role in immersive VR technology by allowing for a natural viewing experience of a digital environment. However, besides psychological components, other components that would address additional senses, such as touch or smell, might further enhance the experience and make it even more natural^23^. Additional components of immersive VR that address different senses are still in their infancy, though. Maximizing the technological support for creating immersion, supporting the mental transition into another, non-human being is one of the challenges on the road to creating impactful experiences that lead to nature relatedness, and, by that to behavioural change^1^. The access to such technology is another limiting factor, posing the challenge of finding a balance between technological accessibility and immersive technology. Nevertheless, features such as translating head movements into the positioning and rotation of the camera are already easily supported by low-cost solutions for smartphones, such as Google Cardboard and similar products, opening a large market for immersive educational applications. The experiment could demonstrate that even short applications are able to impact participants. Further research is needed to explore the optimal duration and design of similar applications in order to provoke self-reflective processes and ultimately, nature relatedness. Once the details of optimization are clear, the potential of bringing people all around the globe closer to nature, closer to becoming nature by taking on the perspective of one of its agents in a playful and entertaining manner, can be harnessed to support one of the key components in combatting climate change—human understanding and behaviour.
